# PRAME Gene Expression in Acute Leukemia and Its Clinical Significance

**DOI:** 10.3969/j.issn.2095-3941.2012.01.013

**Published:** 2012-03

**Authors:** Kai Ding, Xiao-ming Wang, Rong Fu, Er-bao Ruan, Hui Liu, Zong-hong Shao

**Affiliations:** Department of Hematology, General Hospital of Tianjin Medical University, Tianjin 300052, China

**Keywords:** preferentially expressed antigen of melanoma, gene, acute leukemia, minimal residual disease, immunotherapy

## Abstract

**Objective:**

To investigate the expression of the preferentially expressed antigen of melanoma (PRAME) gene in acute leukemia and its clinical significance.

**Methods:**

The level of expressed PRAME mRNA in bone marrow mononuclear cells from 34 patients with acute leukemia (AL) and in 12 bone marrow samples from healthy volunteers was measured via RT-PCR. Correlation analyses between PRAME gene expression and the clinical characteristics (gender, age, white blood count, immunophenotype of leukemia, percentage of blast cells, and karyotype) of the patients were performed.

**Results:**

The PRAME gene was expressed in 38.2% of all 34 patients, in 40.7% of the patients with acute myelogenous leukemia (AML, *n*=27), and in 28.6% of the patients with acute lymphoblastic leukemia (ALL, *n*=7), but was not expressed in the healthy volunteers. The difference in the expression levels between AML and ALL patients was statistically significant. The rate of gene expression was 80% in M_3_, 33.3% in M_2_, and 28.6% in M_5_. Gene expression was also found to be correlated with CD15 and CD33 expression and abnormal karyotype, but not with age, gender, white blood count or percentage of blast cells.

**Conclusions:**

The PRAME gene is highly expressed in acute leukemia and could be a useful marker to monitor minimal residual disease. This gene is also a candidate target for the immunotherapy of acute leukemia.

## Introduction

The PRAME gene was discovered by Ikeda and his colleagues in a melanoma patient in 1997 ^[^^1^^]^. This gene, which is located in 22q11 of the human chromosome and codes for a protein with 509 amino acids, is a tumor antigen recognized by HLA-24 and afterward presented to cytotoxic lymphocytes (CTL). In normal tissues, PRAME is expressed in the testis, adrenal gland, ovary, and endometrium, and its expression levels are lower by more than 3 logarithmic series compared with that in tumor tissues ^[^^2^^]^. Studies abroad suggest that the PRAME gene is highly expressed in leukemic cells ^[^^3^^]^, and its expression levels are correlated with the relapse and the remission of leukemia ^[^^4^^]^.

Continuous gene expression monitoring during treatment could determine the effects of chemotherapy and whether leukemic cells are drug resistant ^[^^2^^]^. Until now, however, local investigations and data on PRAME gene are rare. Hence, this study investigated PRAME mRNA expression in leukemic patients and its relationship with clinical data such as white blood cell count, bone marrow blast count, immunophenotype of leukemia, and karyotype. This study also presents theoretical values to monitor minimal residual disease (MRD) and the immunotherapy of leukemia.

## Patients and Methods

### Patients

Written informed consent was obtained from all patients before the trial. A total of 34 leukemia patients (according to FAB diagnostic criteria) were enrolled in the study, including 24 newly diagnosed and 10 remission patients. Fifteen patients were male, and 19 were female. Their median age was 42.5 years (ranged from 9 to 72 years). Twenty-seven patients had acute myelogenous leukemia) (AML): 12 M_2_ cases (8 de novo, 4 remission), 5 M_3_ cases (1 de novo, 4 remission), 7 M_5_ cases (6 de novo, 1 remission), and 1 M_4EO_ case. Seven had acute lymphoid leukemia (ALL) (3 children and 4 adults). Twelve healthy volunteers were enrolled as control.

### Methods

#### Extraction of mononuclear cells

Bone marrow samples, 1 mL each, were obtained and anti-coagulated with 2% EDTA, dealed with 30 mL hemolysin (1:10 diluted), and then stored in a dark place for 10 min. The samples were centrifugated at 3,000 r/min for 10 min, and the sediments obtained were washed with PBS. The sediments were bone marrow mononuclear cells.

#### Extraction of total RNA

(1) Mononuclear cell lysis. Into each PCR tube with 10^6^ cells, 10 µL lysis buffer and 0.72 µL β mercaptoethanol were added, and the resulting mixtures were mixed for 5 min.

(2) Liquid phase separation. Into each of the mixtures from step 1 was added 10 µL sodium acetate (2 mmol/L). The resulting mixtures were mixed well and allowed to stand for 5 min. Then 100 µL phenol water was added, and again, the mixtures were mixed well and allowed to stand for 5 min. Finally, 20 µL chloroform/isoamyl alcohol was added, and the mixtures were mixed well and centrifugated at 12,000 r/min at 4°C for 5 min. Supernatants were collected.

(3) RNA precipitation. Into the supernatants was added 100 µL isoamyl alcohol. The resulting mixtures were mixed well, placed on ice for 10 min, and centrifugated at 12,000 r/min at room temperature for 5 min. The sediments obtained were the total RNA.

(4) RNA washing (performed twice). Into the sediments was added 75% ethanol (diluted with DDH_2_O). The resulting mixtures were mixed well and then centrifugated at 12,000 r/min at room temperature for 5 min. Sediments were collected.

(5) Redissolving RNA. The sediments were dried and then dissolved with DEPC-H_2_O in a water bath. Purity was determined via absorbance scanning (photometric value, 1.8 to 2.0).

#### RT-PCR

(1) Primers. PRAME: upstream primer – 5’-CTG TAC TCA TTT CCA GAG CCA GA- 3’; downstream primer – 5’-TAT TGA GAG GGT TTC CAA GGG GTT-3’; the amplified fragment length had 561 bp. β-actin (internal control): upstream primer – 5’-ATC TGG CAC CAC ACC TTC TAC AAT GAG CTG CG-3’; downstream primer – 5’-CGT CAT ACT CCT GCT GAT CCA CAT CTC-3’; the amplified fragment length was 800 bp.

(2) Reverse reaction. Reaction systems: sample total RNA, 1 µg to 5 µg; DDH_2_O, 11 µL; Oligo-p(dT)18, 1 µL; 5×reaction buffer, 4 µL; RNase inhibitor (20 U/µL), 1 µL; dNTP, 2 µL; and M-MuLV reverse transcriptase, 1 µL. The mixtures were incubated at 37°C for 60 min and then at 70°C for 10 min.

(3) PCR amplification. Reaction systems: cDNA, 1 µL 2 × PCR Master 25 µL; DDH_2_O, 22 µL; and upstream and downstream primers, 1 µL each. PRAME gene amplification conditions: 94°C 5 min, (94°C 1 min, 64°C 50 s, 70°C 1 min) × 34 cycles. β-actin amplification conditions: 94°C 4 min, (94°C 30 s, 68°C 1 min, 72°C 1 min × 30 cycles. (4) Product analysis. The PCR products (8 µL) were subjected to 2.5% agarose gel electrophoresis at 120 V for 30 min and then analyzed under ultraviolet lamp.

### Statistical analysis

SPSS13.0 analysis software was used. Single-factor ANOVA, homogeneity of variance analysis, and two-sample *t* test were performed.

## Results

### PRAME gene expression in patients with acute leukemia (AL) (Table 1)

**Figure 1:**
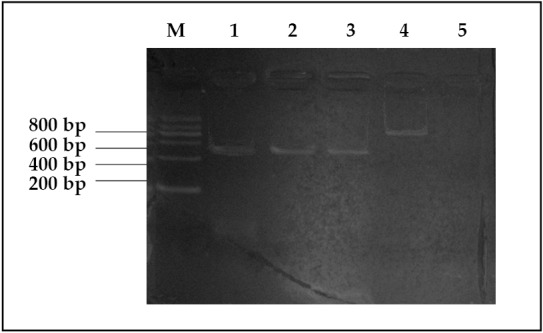
Electrophoretic pattern of PRAME mRNA. M, Marker; 1, M_2_; 2, M_3_; 3, ALL; 4, β-actin; 5, negative control.

In the 34 leukemia patients, the expression rate of the PRAME gene was 38.2% (*n*=34) (**Figure 1**). In the AML patients, the expression rate was 40.7% (*n*=27), and in the de novo AML patients, the expression rate was 43.75% (*n*=16). In the ALL patients, the expression rate was 28.6% (*n*=7). The expression rate of the PRAME gene was higher in the AML patients than in the ALL patients (*P*>0.05).

**Table 1:** PRAME positive patients with different types of AL.

FAB classification	Positive	Negative	Positive rate (%)
AML	11	16	40.7
M_2_ (de novo)	3	5	37.5
M_2_ (remission)	1	3	25
M_3_(de novo)	1	0	100
M_3_ (remission)	3	1	75
M_4EO_(de novo)	0	1	0
M_5_(de novo)	2	4	33.3
M_5_(remission)	0	1	0
CML-AML	1	0	
CMML-AML	0	1	
ALL(de novo)	2	5	28.6
Children	1	2	33.3
Adults	1	3	25

AML, acute myelogenous leukemia; ALL, acute lymphoid leukemia.

### Correlation between PRAME gene expression and clinical data

The expression of the PRAME gene did not have an obvious relationship with patients’ gender, age, or white blood cell count. Gene expression was found higher in the M_2_ and M_3_ groups than in the M_5_ and ALL groups (*P*<0.05).

In the AML patients, the gene expression was higher in M_3_ (80%) than in M_2_ (33.3%) (*P*<0.05). The study also investigated the percentage of blast or blast+immature cells in the bone marrow of the patients; no correlation was found between this factor and PRAME gene expression. The fluorescence intensity of the specific surface markers of the leukemic cells, including CD3, CD5, CD7, CD10, CD13, CD14, CD15, CD19, CD20, CD22, CD33, CD34, CD64, and CD117, was measured, and correlation analysis between fluorescence intensity and PRAME gene expression was performed. Results showed obvious differences in the expression of CD15 and CD33 between the PRAME-positive and negative groups (**Table 2** and **Table 3**).

**Table 2:** PRAME gene expression and CD15.

PRAME expression	Sample number	CD15 mean value^a^	Standard error
Positive	8	73.48%	3.12%
Negative	9	82.18%	2.54%

*t*-test, *P*<0.05; ^a^, the mean value is the average fluorescence intensity in flow cytometry.

**Table 3:** PRAME gene expression and CD33.

PRAME expression	Sample number	CD33 mean value^a^	Standard error
Positive	9	52.98%	3.08%
Negative	10	35.00%	3.88%

*t*-test, *P*<0.005; ^a^, the mean value is the average fluorescence intensity in flow cytometry.

Of the 34 leukemia patients, 27 underwent chromosome examination. Thirteen patients had karyotype abnormality, including gene shifts and deletions. Of the 12 healthy volunteers, 4 underwent chromosome examination, in which no chromosomal aberration was found. Moreover, PRAME expression was observed to be higher in the patients with abnormal karyotypes than in those with normal ones (**Table 4**).

**Table 4:** PRAME gene expression and karyotypic abnormality.

Karyotype	PRAME gene positive	PRAME gene negative
Normal	4	10
Abnormal	8	5

χ^2^ test, *P*<0.05

### Follow-up

In this study, PRAME gene expression in all patients was continuously monitored before every cycle of chemotherapy. In one patient with ALL, PRAME gene expression disappeared after the first cycle of chemotherapy, and this improvement was accompanied by complete morphological remission (complete remission; CR). Very interestingly, in an M_2_ patient who had CR, PRAME gene expression became positive and thus indicated the risk of relapse. Three months later, a small group of blast was found in this patient via flow cytometry (FCM) examination. Then after five months, the patient morphologically relapsed.

## Discussion

Until now, the criteria for the clinical remission of leukemia are based on symptoms, physical signs, blood cell count, and percentage of blast and immature cells in the bone marrow. According to these criteria, even patients with CR still have some leukemic cells which cannot be detected by common microscopy. These cells are called MRD. MRD is the primary cause of leukemia relapse. Clearly, in the early stages of leukemia or after CR, the means of monitoring and the eradication of leukemic cells are critical in the treatment of the disease. PCR, a modern biological method, has high sensitivity that can reach up to 10^-4^ to 10^-6^, which is better than that of FCM or fluorescence in situ hybridization. However, the use of PCR in leukemia is limited because only several types of leukemia have specific gene markers such as PML/RARa, AML1/ETO, and bcr/abl. As a result, finding a widely expressed targeting gene is a precondition for the early diagnosis, MRD monitoring, and immunotherapy of leukemia.

The expression of PRAME was higher in this study than that reported by Paydas ^[^^4^^]^ and Baren ^[^^5^^]^, but was lower than that reported by Steinbach ^[^^6^^]^. These variations may have been caused by the differences in the subjects examined. This study included both adults and children, Paydas ^[^^4^^]^ and Baren^[^^5^^]^ focused on adults with acute leukemia, and Steinbach^[^^6^^]^ focused on children with acute leukemia. From the subtypes of AML, M_3_ patients had the highest expression rate (80%), followed by M_2_ (33.3%), and then M_5_ (28.6%), similar to the results of Paydas ^[^^4^^]^ and Baren ^[^^5^^]^. Like the findings of other studies, no PRAME expression was found in the 12 healthy volunteers. To further investigate whether PRAME gene expression is correlated with the clinical characteristics of leukemia, Paydas ^[^^4^^]^ examined 74 patients, including 68 patients with de novo acute leukemia, 3 with chronic myeloid leukemia-blastic phase, and 3 with myelodysplastic/myeloproliferative syndrome-blastic transformation. Their results suggested that PRAME gene expression was not correlated with age, gender, size of peripheral lymph nodes, hemoglobin and white blood cell count, platelet count, LDH level, ALP level, albumin level, cell surface antigen, or response to therapy. This study also concluded that PRAME expression was not correlated with age, gender, white blood cell count, or percentage of blast and immature cells in the bone marrow.

Several studies have demonstrated that the expression of the PRAME gene is correlated with chromosome translocation ^[^^5^^,^^7^^]^. This study also proved that PRAME was somehow related to chromosome abnormality. However, whether the PRAME gene is caused by chromosome changes needs further investigation. Moreover, this study found that PRAME gene was correlated with CD15 and CD33, similar to the results of Paydas ^[^^4^^]^ and other related studies.

Compared with Wilm’s tumor (WT1) gene, PRAME gene, as another widely expressed leukemia gene, is more widely expressed in patients with normal karyotypes. According to Matsushima ^[^^2^^]^, WT1 can only be detected when the titer of leukemic cells is above 10^-3^ to 10^-4^. For PRAME, however, the detected titer is lower than 10^-4^. Furthermore, WT1 is mainly expressed in AML patients. In APL and ALL patients, the WT1 gene is not expressed or is detected at titers lower than 10^1^ to 10^2^ copies/mg RNA. By contrast, PRAME gene is highly expressed in all these types. These findings suggest that the combined use of WT1 and PRAME can improve the detection of MRD ^[^^8^^]^. In some diseases, the expression of PRAME gene becomes positive earlier, but disappears later, than that of WT1. Hence, PRAME may be a better candidate than WT1 for MRD monitoring. Through dynamic monitoring of PRAME gene in one of the patients examined, this study found the existence of MRD 3 months earlier than FCM and 5 months earlier than morphological relapse. This particular case proved the value of detecting PRAME gene expression in monitoring MRD. Moreover, the quantitative examination of the PRAME gene can be used to tailor chemotherapy schedules for individual leukemia patients: when the level of the PRAME gene decreases to normal, patients do not need to have chemotherapy; however, when the expression level of PRAME increases rapidly, patients must be treated with appropriate chemotherapy. This practice is different from current chemotherapeutic strategies guided by morphological changes.

The function of PRAME gene and its protein is not yet clear. This study found that the expression of PRAME gene was much higher in patients with chromosomal abnormalities than in those with normal chromosomes. Furthermore, chromosomal abnormality always leads to the emergence of fusion genes and proteins, which often participate in intracellular signaling pathways. Thus, speculating that PRAME protein participates in intracellular signaling pathways is reasonable. Some studies have demonstrated that the PRAME protein is an inhibitor of the RARa pathway^[^^9^^,^^10^^]^. Assisted by retinoid acid (RA), the PRAME protein combines with RAR, and the resulting complex inhibits ligand-mediated receptor activation. Through chemotaxis for polycomb protein, the RAR element-mediated downstream initiation of transcription is inhibited. As a result, cell differentiation and apoptosis are halted. A recent study also suggests that the PRAME protein is a subunit of ubiquitin ligase, and it participates in the activation of the nuclear factor promoter Y ^[^^11^^]^.

The PRAME gene is also a promising component in the immunotherapy of leukemia. As a cell membrane protein, the PRAME, presented by HLA-24, can be recognized and lysed by CTL. Hence, PRAME specific CTLs can be extracted and cultivated from melanoma patients. PRAME protein is also not expressed or is expressed only at very low levels in normal tissues, and PRAME mRNA is not expressed in CD34 cells ^[^^12^^]^. Therefore, PRAME is a feasible candidate targeting antigen for tumor immunotherapy or vaccines. A recent study has proven that PRAME protein has antigenicity *in vitro*, and stimulates the proliferation and activation of specific CD8^+^ CTL ^[^^13^^]^.

In summary, PRAME gene is a widely expressed leukemia gene that has been the focus of much scientific research in recent years. This gene is not only widely expressed in many hematological malignant diseases but is also intimately related to the progression and the remission of diseases. The hypothesis that the PRAME gene can be used in the monitoring of MRD is gradually being accepted, and the determination of this gene’s function is paving a promising way for the immunotherapy and gene-targeting treatment of leukemia.
